# Post-Surgical Central Nervous System Infections in the Era of Multidrug Antibiotic Resistance in Greece—A Single-Center Experience of a Decade

**DOI:** 10.3390/pathogens14040390

**Published:** 2025-04-16

**Authors:** Konstantinos Markakis, Konstantina Kapiki, Angela Ava Arbelle Edric, Asimina Aphrodite Pappas, Georgios Feretos, Sideris Nanoudis, Dimitrios Pilalas, Theodoros Michailidis, Efthymia Protonotariou, Lemonia Skoura, Nikolaos Foroglou, Symeon Metallidis, Olga Tsachouridou

**Affiliations:** 1 Infectious Diseases Unit, 1st Internal Medicine Department, AHEPA University Hospital, School of Medicine, Aristotle University of Thessaloniki, S. Kiriakidi Str. 1, 54636 Thessaloniki, Greece; conmark@windowslive.com (K.M.); kwnnakap@gmail.com (K.K.); aarbelle@auth.gr (A.A.A.E.); aap249@scarletmail.rutgers.edu (A.A.P.); sidnanoudis@yahoo.gr (S.N.); pilalas_jim@hotmail.com (D.P.); thgmichailidis@gmail.com (T.M.); metallidissimeon@yahoo.gr (S.M.); 2 Department of Neurosurgery, AHEPA University Hospital, School of Medicine, Aristotle University of Thessaloniki, S. Kiriakidi Str. 1, 54636 Thessaloniki, Greece; g.feretos@gmail.com (G.F.); nforoglou@auth.gr (N.F.); 3 Department of Microbiology, AHEPA University Hospital, School of Medicine, Aristotle University of Thessaloniki, S. Kiriakidi Str. 1, 54636 Thessaloniki, Greece; protonotariou@gmail.com (E.P.); mollyskoura@gmail.com (L.S.)

**Keywords:** post-surgical central nervous system infections, Gram-negative bacteria, external ventricular drain, *Acinetobacter baumannii*, mortality, post-surgical meningitis, ventriculitis

## Abstract

Post-surgical central nervous system infections (PCNSIs) are a major cause of morbidity, poor functional outcomes and mortality in neurosurgical patients. These infections complicate operations of the CNS or are related to the use of neurosurgical devices or drainage catheters. Gram-negative bacteria, with multiple resistance patterns, are often isolated and these infections are difficult to treat, due to suboptimal antibiotic therapeutic levels in the cerebrospinal fluid (CSF). This is a retrospective study of PCNSIs between 2014 and 2024 in a single center of a tertiary hospital in Thessaloniki, Greece. Out of 2401 neurosurgical procedures, forty-one were complicated by PCNSIs, yielding a total PCNSI prevalence of 1.7%. Thirty-five involved cases with positive CSF culture. The most common interventions were craniotomies for the resection of tumors or other lesions (30.1%). Most cases referred to an EVD infection. *Acinetobacter baumannii* was the most commonly isolated pathogen (34.1%), followed by coagulase-negative *Staphylococcus* (22%) and *Pseudomonas* spp. (14.6%). Colistin and tigecycline were the most prescribed combination regimens. The median time to the first positive CSF culture postoperatively was 11 days (IQR 18 days). Empirical antibiotic treatment was adequate in 26 (63.4%) cases. The mortality rate among these patients was 65.7%. Survivors were significantly younger than non-survivors (*p* < 0.01) and had a shorter ICU length of stay (*p* < 0.01). The type of infection, time to infection onset, isolated pathogen, susceptibility to the empirical treatment and Charlson Comorbidity Index did not differ between the two groups. The mortality rate remains high in patients with PCNSIs. An integrated approach including surgical source control, supportive care, combination antimicrobial therapy and subsequent rehabilitation are mandatory to achieve treatment success and neurological convalescence.

## 1. Introduction

Post-surgical central nervous system infections (PCNSIs) are a major cause of morbidity and mortality in neurosurgical patients. PCNSIs include post-surgical meningitis (PM), ventriculitis, subdural empyema (SE), brain abscess (BA) and spinal abscess. PM is defined as the inflammation of the meninges caused by bacteria, viruses, fungi, parasites and non-infectious factors which occurs after a neurosurgical procedure. Diagnosis usually occurs within a month post-surgery; however, a PCNSI can occur months or even years post intervention [1,2,3,4]. Due to the increasing frequency of foreign body implantation following neurosurgical procedures—like the placement of cerebrospinal fluid (CSF) shunts or external ventricular drains (EVD), intrathecal pumps and deep brain stimulators —an increase in PM incidence has been observed [5].

Shunt infections can arise through the colonization of the shunt by skin flora. The colonization occurs either during the initial placement of the shunt, perioperatively due to non-sterile technique or postoperatively through skin or mucosa defects. Furthermore, direct contamination of the distal end of the shunt lying in the intraperitoneal cavity, the pleural or vascular space, or hematogenous seeding can also lead to PCNSIs [6]. An infection rate of about 4–17% has been reported in patients with shunts [3]. The majority of CSF shunt and EVD infections are caused by bacteria of the skin flora, with coagulase-negative staphylococci followed by *S. aureus* and *Cutibacterium acnes* being the most common [1]. Further microorganisms implicated in shunt infections include *streptococci*, diphtheroid organisms such as *Corynebacterium jeikeium*, Gram-negative bacteria (such as *Acinetobacter baumannii, Pseudomonas aeruginosa* and others), anaerobes, mycobacteria and fungi [3,7,8]. In neurosurgical procedures, the formation of bacterial biofilm following the contamination of foreign bodies seems to play an essential role in the promotion of PCNSIs [6].

Nevertheless, besides the type of surgery and the underlying condition of the patient, the local microbial epidemiology also seems to play a role in the incidence of the various pathogens implicated. Characteristically, in a Greek cohort of 334 patients who underwent craniotomy, Gram-negative bacteria were responsible for the majority of the cases, being isolated in 88% of the PCNSIs [9], while in an Italian study, Gram-positive and Gram-negative bacteria were equally represented in cultures [10].

A series of risk factors for PCNSIs have been identified. These include CSF leakage, CSF communication with the environment, incision infection, male sex, perioperative corticosteroid use, malignancy, duration of surgical procedure, previous brain infection, craniectomy with delayed cranioplasty, length of intraventricular catheter placement, ventriculostomy and extraventricular drainage [11,12,13,14,15,16,17]. The data on the role of prophylactic administration of antibiotics are contradictory, with some studies demonstrating infection rate decline whereas other studies do not [11,12,18]. Furthermore, some studies supported that antibiotic prophylaxis not only lacks benefit, but on the contrary can be harmful, leading to the emergence of multidrug-resistant bacteria in neurosurgical patients [11,12].

The diagnosis of PCNSIs is challenging [19]. Clinical signs and symptoms are often absent or non-specific and the available tests insensitive [4,20]. Distinguishing between simple shunt colonization and meningitis caused by skin flora bacteria is a real challenge when the infection focus is not clear. Procalcitonin [21], CSF lactate levels and, more recently, next-generation sequencing have been recognized as valuable diagnostic procedures which assist to differentiate between PCNSIs and post-surgical CNS inflammation [5]. Prolonged hospitalization, often in an intensive care unit, increases the risk of bacterial colonization and invasive infections, like PCNSIs, with multidrug-resistant bacteria and fungi. Thus, antimicrobial resistance is often encountered and forms a major problem for their management [22]. In addition, many of the available antibiotics cannot sufficiently penetrate the blood–brain barrier, thus not achieving therapeutic drug levels in the CSF [19]. Lastly, the presence of foreign bodies further complicates the successful treatment of PCNSIs, often making the extraction of the device unavoidable.

The incidence of PCNSIs varies among different cohorts and the type of surgery, with reports of rates varying from 1% to over 7% [23,24]. This large variation in the reported incidence could also be attributed to the difficulty in the development of a universal definition of PCNSIs. Recently, a meta-analysis, which included over 30,000 patients who underwent an elective neurosurgical procedure, reported an incidence rate of PM of 1.6% [25]. As to the outcomes, large fluctuations of the mortality rate of PCNSI have been reported. In a cohort of 52 cases of PM, the mortality rate was 8% [26]. However, other studies recorded a mortality rate which exceeded 30% [27]. PCNSIs after spinal surgery are less common than cranial surgery and usually milder. In a cohort of over 20,000 patients who underwent spinal surgery, PCNSI was diagnosed in merely 21 cases [28].

The objective of this retrospective study is to analyze and characterize the clinical and microbiological features of patient cases with postoperative meningitis caused by multidrug-resistant bacteria. By reviewing a large series of patient records, the study aims to identify patterns in the incidence, risk factors, treatment outcomes, and complications associated with these infections. The findings of this study provide insights into the local epidemiology of these severe infections, the efficacy of current therapeutic approaches and will contribute to the development of more effective strategies for managing and preventing postoperative meningitis in the context of multidrug-resistant pathogens.

## 2. Material and Methods

### 2.1. Study Population

A retrospective study was performed on patients diagnosed with PCNSIs, predominantly PM, between 2014 and 2024. The study was carried out at AHEPA University Hospital of Thessaloniki, Northern Greece, which hosts an academic neurosurgery clinic. All the cerebrospinal fluid (CSF) cultures registered in the Clinical Microbiology Laboratory were reviewed. The diagnostic criteria for a PM case in the literature vary. In line with previous studies, we adopted the nosocomial infections surveillance definition [29].

The medical chart of each patient was reviewed, and epidemiological and clinical data were collected: age, sex, indication for surgery, type of infection (ventriculitis, meningitis, epidural abscess, subdural empyema, brain abscess), time elapsed since the intervention, the isolated pathogen, the presence of multibacterial infections, the duration of empirical and targeted treatment, days in ICU, outcome, comorbidities, concomitant infections and relevant CSF and blood parameters. Previous antibiotic treatment was defined as antibiotic administration for at least 48 h during the 3 months before the meningitis diagnosis.

A prophylactic perioperative dose of vancomycin plus ceftriaxone was administered intravenously in each included patient perioperatively according to local guidelines. CSF samples were obtained with the use of an intraventricular catheter, if present, or lumbar puncture.

### 2.2. Microbiology Data

Bacterial identification and antimicrobial susceptibility testing were performed using the automated system VITEK2 (bioMérieux, Marcy l’Etoile, France). Susceptibility testing results were interpreted according to the EUCAST breakpoints. The Biofire^®^ FilmArray^®^ meningitis/encephalitis panel (bioMérieux, Marcy l’Etoile, France) employed for rapid molecular diagnosis was available in our hospital at the time and was used instantly. For MDR Gram-negative bacteria, all isolates were tested phenotypically for the detection of metallo-β-lactamases (MBL), *Klebsiella pneumoniae* carbapenemase (KPC), or both categories, using the meropenem disc test.

The Institutional Review Board of the Hospital has approved this study (Protocol No: 24359/17 June 2024).

### 2.3. Statistics

Data were analyzed for Gaussian distribution (Kolmogorov–Smirnov test). In the case of normal distribution, data are presented as mean with standard deviation (SD), otherwise as median with interquartile range (IQR). The t-test was performed for parametric and Mann–Whitney U test for non-parametric variables. Pearson correlation analyses for normal variables and Spearman correlation analyses for non-parametric variables were performed to assess the association between the different epidemiological characteristics of the patients, the antibiotic treatment, isolated bacteria and laboratory findings and the outcomes (death, ICU length of stay). The chi-square test was performed to compare categorical data between groups. *p* < 0.05 was regarded statistically significant. All statistical analyses were performed using SPSS Statistics 26 (SPSS Inc., Chicago, IL, USA).

## 3. Results

### 3.1. Demographic Characteristics

A total of 2401 neurosurgical procedures were performed in the study time period. The most common was craniotomy for resection of tumors or other lesions (30.1%), followed by procedures which included burr holes, aspiration or drainage (16.1%), craniotomies for non-oncologic indications (hemorrhage drainage, hematoma evacuation or clipping) (14.2%), EVD placement (9%), VP shunt placement (7.2%), transnasal or transsphenoidal procedures (5.5%), craniectomies (3.8%), brain biopsies (2.1%), Ommaya reservoir implantations (0.1%) and other neurosurgical procedures not further specified.

In 41 patients, the neurosurgical procedures were complicated with PCNSIs, yielding a total PCNSI prevalence of 1.7% over the examined time period. These cases of PCNSIs involved, in total, 35 patients with positive CSF cultures. Seventy-four percent of the patients were male. The median age at operation was 54 years (IQR 22). The mean ICU length of stay was 21.8 (SD 19) days. Among the patients included, 8 (22.9%) carried a VP shunt and 23 (65.7%) an EVD at admission or during their hospitalization. The most frequent underlying diseases were neoplasm (34%) and hemorrhage (46%), followed by hydrocephalus (8.5%). As to the type of infection, an EVD infection was diagnosed in 24 cases, PM in 7, a VP shunt infection in 6, PV in 4 and a trauma-related infection in 3 cases.

CSF examination revealed the following findings: median white blood cell (WBC) count in CSF was 138 cells/mm^3^ (IQR 702 cells/mm^3^), protein was 95 mg/dL (IQR 205.6 mg/dL) and median glucose level in CSF was 40 mg/dL (IQR 48). The median blood WBC count was 10,510 cells/μL (IQR 9080 cells/μL).

**Figure 1:**
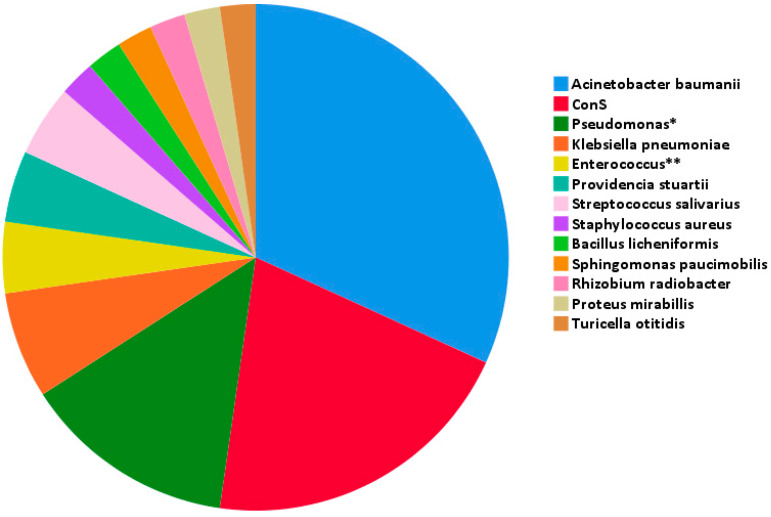
Isolated pathogens in CSF. Abbreviations: ConS: coagulase-negative Staphylococcus; CSF: cerebrospinal fluid; *: 4 Pseudomonas aeruginosa, 1 Pseudomonas putida, 1 Pseudomonas fluorescens; **: 1 Enterococcus faecalis and 1 Enterococcus faecium cases.

A refractory CNS infection was diagnosed in six patients. Three of the patients were diagnosed with polymicrobial CNS infection. The most common pathogen was *Acinetobacter baumannii,* which was isolated in 14 cases (34.1% of total infections), followed by coagulase-negative *Staphylococcus* (ConS) in 9 cases (22% of cranial infections) and *Pseudomonas (Ps. aeruginosa, Ps. putida* and *Ps. fluorescens)* in 6 patients (14.6%), *Klebsiella pneumoniae* in 3 cases (7.3%), and *Enterococcus (E. faecalis* and *E. faecium)*, *Providencia stuartii* and *Streptococcus salivarius* in 2 patients (4.9%), respectively. *Bacillus* licheniformis, *Proteus mirabilis*, *Rhizobium radiobacter*, *Staphylococcus aureus*, *Sphingomonas paucimobilis* and *Turicella otitidis* were isolated in one patient each (2.4%) Figure 1. A detailed list of the pathogens encountered and associated interventions is provided in Table 1.

The resistance patterns of the most commonly isolated pathogens are summarized in Table 2.

Regarding the empirical antibiotic treatment patients received while cultures were pending, ten (24.4%) received empirical antibiotic monotherapy. Three (7.3%) of them were treated with meropenem, one (2.4%) with ampicillin, one (2.4%) with ampicillin/sulbactam, one (2.4%) with ceftaroline, one (2.4%) with cefepime, one (2.4%) with colistin, one (2.4%) with linezolid and one patient (2.4%) with vancomycin. The rest of the patients (31, 75.6%) initially received combinations of antibiotic regimens. Colistin and tigecycline were the most commonly prescribed regimens as part of combination therapy, with 20 (48.8%) and 14 (34.1%) patients each. Meropenem was administered as part of a combination treatment in 12 (29.3%) patients, linezolid also in 12 (29.3%) patients, vancomycin in 8 (19.5%) patients, piperacillin/tazobactam in 4 (9.8%) patients, ceftazidime/avibactam was administered in 3 (7.3%) patients, and fosfomycin in 2 (4.9%) patients. Lastly, amikacin, cefepime, ceftriaxone, daptomycin, levofloxacin, vancomycin were administered as part of combination antibiotic therapy in one patient each (2.4%). One patient also received fluconazole as part of the initial empirical treatment (2.4%). The median time to the first positive CSF culture postoperatively was 11 days (IQR 18 days) and the median duration of empiric antimicrobial therapy was 7 days (IQR 7 days).

The empirical antibiotic treatment was deemed adequate in 26 (63.4%) patients once the CSF cultures results were available, whereas in the rest of the patients (36.6%), the isolated pathogen was not susceptible to the administered antibiotic regimens. Fourteen patients were diagnosed with another nosocomial infection besides PCNSIs during their hospitalization, with urinary tract infections (10, 28.6%) and lower respiratory tract infections (9, 25.7%) being the most common.

### 3.2. Outcomes

Of the 35 patients with positive CSF cultures included in the study, 12 (34.3%) were discharged and 23 (65.7%) died during their hospital stay. In 12 patients with an EVD and in 6 patients with a VP shunt, the foreign body had to be removed or replaced during their hospital stay as a result of the PCNSIs.

### 3.3. Survivors Vs. Non-Survivors

Survivors were significantly younger than non-survivors (*p* < 0.01) and had a shorter ICU length of stay (*p* < 0.01), as shown in Table 3. The type of infection, isolated pathogen and susceptibility to the empirical treatment, Charlson Comorbidity Index, previous CNS surgery, hospitalization and other comorbidities did not differ between the two groups. Furthermore, while non-survivors had a more delayed onset of post-surgical infection (17 days vs. 10 days), this was not significant for the outcome. Compared to survivors, non-survivors received vancomycin as part of the empirical antibiotic treatment significantly less often (*p* = 0.038). The usage rate of other antibiotics did not differ between survivors and non-survivors. No differences in the CSF findings between the two groups were noted. Interestingly, survivors had a higher median procalcitonin level than non-survivors (*p* = 0.015); however, the sample size was small.

### 3.4. ICU Length of Stay

ICU length of stay was significantly higher in patients who received colistin (*p* < 0.01), or tigecycline (*p* < 0.01) as part of the initial empirical antibiotic treatment combination. On the contrary, infection with *ConS* (*p* < 0.01), use of vancomycin (*p* < 0.01), previous hospitalization (*ρ* = 0.584; *p* < 0.001) and previous administration of antibiotics for other infections (*ρ* = 0.611; *p* < 0.001) were related with a shorter ICU length of stay. Lastly, the duration of antibiotic treatment (*ρ* = 0.337; *p* = 0.038) correlated with ICU length of stay.

## 4. Discussion

To the best of our knowledge, this is the largest study so far assessing the incidence, epidemiological and microbiological characteristics of PCNSIs in Greece, an endemic country for Gram-negative multidrug-resistant pathogens. Most data available derive from case reports [30] and case series [31] of difficult-to-treat CNS infections.

The microorganisms responsible for PM are part of the patient’s skin flora (*Staphylococcus* spp., *Cutibacterium acnes*) or have a nosocomial origin. Overall, colonization precedes infection [32]. Regarding EVDs, most drains develop biofilm usually seven days post-insertion [33]. Apart from Gram-positive, Gram-negative pathogens, particularly *A. baumannii*, are related to biofilm-associated nosocomial infections [34,35].

The relative frequency of the isolated pathogens varies between countries or hospital environments [36]. In a recent multicenter study, both Gram-positive and Gram-negative pathogens were isolated equally from CSF cultures [10]. However, there has been a steady increase in the prevalence of difficult-to-treat isolates in many centers around the globe, including multidrug-resistant *Pseudomonas aeruginosa*, and the carbapenemase-resistant *A. baumannii* and *Klebsiella pneumoniae* [37,38,39]. It should be highlighted that irrespective of the isolated pathogen (Gram-positive vs. Gram-negative), there were no consistent differences regarding outcomes [40,41].

As expected, the vast majority of the PCNSIs recorded were attributed to multidrug-resistant pathogens, with *A. baumannii* being the most commonly isolated pathogen in CSF cultures. These microbiological data are in accordance with previous studies regarding PCNSIs in Greek and Italian hospitals, where Gram-negative bacteria (*Acinetobacter* spp., *Klebsiella* spp., *Pseudomonas* spp. and others) formed the majority of the isolated causative pathogens [9,10]. The predominance of Gram-negative bacteria in these countries could be partly explained by the increased incidence of multidrug-resistant strains in those regions, as reported by antimicrobial resistance surveillance in Europe [42]. Similarly, the majority of PCNSIs in an Indian cohort was also attributed on Gram-negative bacteria [43]. In contrast, in several other studies, Gram-positive bacteria, particularly *Staphylococcus* spp., were the predominant pathogens isolated in PCNSIs [18,23,44,45,46].

In the examined population, 1.7% of the neurosurgical procedures was complicated by PCNSIs. The reported prevalence of PCNSIs in previous studies varied between 1.2% in resource-rich and 2.7% in resource-poor or middle-income countries [25].

Post-surgical meningitis is a rare but severe complication following cranial procedures. In nosocomial infections, multidrug-resistant Gram-negative pathogens such as *Acinetobacter baumannii* are often implicated and associated with mortality rates above 40% [47]. The restricted availability of active antimicrobials against carbapenemase-producing *A. baumannii* (CRAB), further complicates the management of CNS infections in terms of tolerability, effectiveness and pharmacokinetics.

The in-hospital mortality burden has increased and varies from 9.3% to as high as 40.3% [7,48]. Settings with multidrug-resistant Gram-negative bacteria are associated with higher mortality [49,50]. However, limited data are available to provide a direct comparison. Apart from mortality and regardless of the causative pathogen, poor neurological outcomes (vegetative state, assisted living) occur in more than 60% of PCNSI patients [7,51]. Of interest, mortality following PCNSIs in a difficult-to treat setting was independently associated with *A. baumannii* isolation and the administration of inappropriate empirical treatment [47,52]. In our study, the overall mortality rate was excessively higher, reaching 60%.

Another alarming fact from this study is that the susceptibility of the isolated pathogens to the initial empirical treatment was 62.3%, although these data did not show differences among survivors and non-survivors. This finding is really interesting. In some studies, it had a positive impact on mortality [47,52]; however, the literature on this field is rather inconclusive. This could be partly explained, initially, by the severity of these infections de novo, regardless of the pathogen or the treatment approach. These results are, however, important to modify our local antimicrobial treatment guidelines, to introduce consultation from an ID specialist to be mandatory and to promote the uptake of initial and surveillance cultures. Factors that may have influenced the results of our study are the relatively small number of PCNSIs, the retrospective study design and the lack of a formal surveillance program for PCNSIs in this surgical department.

In the most recent update of the IDSA nosocomial CNS infection guidelines, the only treatment available for CRAB is colistin, which suboptimally penetrates the CSF [30,53]. Data on efficacy of intravenous antibiotic treatment for CNS infections attributed CRAB are limited. Colistin, however, remains a powerful tool in our armamentarium, especially in countries endemic for *A. baumannii*, like Greece, as also presented in our results. Additionally, the combination of intravenous and intraventricular (IVT) or intrathecal (ITH) administration of colistin in MDR *A. baumannii* ventriculitis/meningitis remains a successful treatment strategy [54] with improved outcomes regarding ICU stay and mortality. Furthermore, colistin is a viable and rather safe option for the systemic antimicrobial treatment of these severe CNS infections with few adverse events [55].

Craniotomy predisposes the patient to infection more than shunt insertion, [56] which is in line with the results of this study. Despite its merit, an EVD comprises a foreign body with subsequent risk of infection. Multiple other risk factors have been identified, like insertion site and technique, prolonged catheterization, presence of blood in the CSF, CSF leak, or regular catheter manipulation and sampling, including EVD change [57].

With regard to the type of infection, the prevalence of infection among patients in which an EVD is used ranges between 0 and 22% [58]. This is in contrast to the data recorded in this study, in which EVD infections comprised the majority of PCNSIs. However, EVD-related infections significantly prolong the duration of hospital stay, and subsequently increase the hospitalization costs and negatively affect functionality and the overall prognosis [58,59]. Previous studies have reported various lengths of stay in hospital for patients with PCNSIs following EVD placement [60,61,62]. Effectively reducing the risk factors of PCNSIs and early identification and treatment is of critical significance to reduce the incidence and mortality rates associated with PCNSIs and to improve the outcomes of patients [63]. In the present study, the duration of hospital stay was prolonged especially in non-survivors and mainly in the ICU, which was comparable to that in the existing literature [60,61,62].

Furthermore, previous studies have shown that longer drainage duration is not a risk factor for developing infection [17,64,65]. On the other hand, other authors reported that the risk of becoming infected is significantly lower after 9 days of EVD [17,63,64,65,66]. Interestingly, other studies have concluded that longer drainage duration implicates the chance of infection development [67,68]. In our study, the median time to infection was 11 days postoperatively, confirming previous studies in which the average time from the operation to the diagnosis of PCNSI was 10.3 days, analogous to the EVD-related infection, which peaked at about 10 days post-insertion [57].

Our study has certain limitations. One main limitation is the retrospective nature of the study, which is associated with possible errors in the collection and interpretation of data. Secondly, the study was conducted in a single center and our findings cannot be generalized. Larger, multicenter studies are needed to gain a deeper insight into the epidemiological and microbiological characteristics of PCNSIs in Greek hospitals.

High morbidity and mortality rates from PCNSIs remain a significant barrier in the management of these infections. An integrated approach including surgical source control, supportive care, combination antimicrobial therapy, and subsequent rehabilitation are mandatory for successful management and neurological convalescence. Novel cerebrospinal fluid biomarkers and molecular microbiology can expedite pathogen identification and treatment [47]. Since limited efficacy data and treatment options are available, especially for multidrug-resistant pathogens, novel treatment options should be applied to further assess the CNS pharmacokinetics [31]. Further prospective studies and surveillance of neurosurgical departments are crucial for the prevention and improvement of PCNSI infection rates.

## Figures and Tables

**Table 1:** Pathogens isolated, types of infection and associated neurosurgical procedures. * *P. aeruginosa* (4 cases), *P. putida* (1 case), *P. fluorescens* (1case). ** *E. faecalis* (1 case), *E. faecium* (1 case).

Pathogen	N	Type of Procedure (No. of Cases)	Type of Infection (No. of Cases)
*Acinetobacter baumannii*	14	EVD placement (6) VP shunt placement and EVD removal (1) Decompressive craniectomy (1) Craniotomy for drainage of hemorrhage (1) Craniotomy for tumor resection (1) Craniotomy for tumor resection and EVD placement (3) Aneurysmal clipping and EVD placement (1)	EVD infection (11) Meningitis (1) Trauma infection (1) Ventriculitis (1)
*ConS*	9	EVD placement (1) EVD replacement (1) VP shunt placement (1) VP shunt chamber puncture (1) VP shunt placement and EVD removal (1) Craniotomy for tumor resection (2) Craniotomy for tumor resection and EVD placement (1) Aneurysmal clipping and decompressive craniectomy (1)	EVD infection (4) Meningitis (3) Shunt infection (2)
*Pseudomonas* spp. *	6	Aneurysmal clipping and decompressive hemicraniectomy (1) Craniotomy for tumor resection (2) Craniotomy for tumor resection and VP shunt placement (1) EVD placement (2)	EVD infection (2) Meningitis (1) Shunt infection (1) Trauma infection (1) Ventriculitis (1)
*Klebsiella pneumoniae*	3	Craniotomy for tumor resection (1) EVD placement (2)	Meningitis (1) EVD infection (2)
*Enterococcus* spp. **	2	VP shunt placement and EVD removal (1) EVD placement (1)	EVD infection (1) Shunt infection (1)
*Providencia stuartii*	2	Craniotomy for tumor resection and EVD placement (1) EVD placement (1)	Trauma infection (1) EVD infection (1)
*Streptococcus* *salivarius*	2	Decompressive craniectomy (2)	Meningitis (1) Ventriculitis (1)
*Bacillus* *licheniformis*	1	EVD placement (1)	Shunt infection (1)
*Proteus mirabilis*	1	Aneurysmal clipping and decompressive hemicraniectomy (1)	Ventriculitis (1)
*Rhizobium radiobacter*	1	EVD placement (1)	EVD infection (1)
*Sphingomonas paucimobilis*	1	EVD placement (1)	EVD infection (1)
*Staphylococcus aureus*	1	VP shunt placement (1)	Shunt infection (1)
*Turicella otitidis*	1	EVD placement (1)	EVD infection (1)

**Table 2:** Resistance patterns of the most commonly isolated pathogens.

	*Acinetobacter*	ConS	*Pseudomonas*	*Klebsiella*	*Enterococcus*	*Providencia*
MDR	2		1	3		1
XDR	9		2		2	
PDR	3					1
MRSE		3				

MDR: multidrug-resistant; XDR: extensively drug-resistant; PDR: pandrug-resistant; MRSE: methicillin-resistant *Staphylococcus epidermidis*; ConS: coagulase-negative Staphylococcus.

**Table 3:** Comparison between survivors and non-survivors.

Characteristics	Survivors	Non-Survivors	*p*-Value
Age (years)	32 (10.8–52.8)	56 (45–65)	<0.01
Sex (male/female)	10/4	19/8	1
Susceptibility to empirical treatment (%)	71.4%	59.3%	0.44
Leukocytes (/mm^3^)	131 (54.5–218)	460 (61–1075)	0.15
Glucose (mg/dL)	44 (24–66)	36 (24–79)	0.7
Protein (mg/dL)	80 (54.4–435.3)	95 (61.3–209.1)	0.82
Leukocytes (/μL)	10,510 (6450–15,800)	10,535 (6665–15,805)	0.83
Creatinine (mg/dL)	0.53 (0.48–0.95)	0.65 (0.39–0.84)	0.35
PCT (ng/mL)	0.57 (0.29–0.81)	0.19 (0.11–0.4)	0.015
ESR (mm/h)	40 (19–40)	22 (16.5–101.7)	0.63
Albumin (g/dL)	2.03 (1.47–3.26)	2.7 (2.27–2.76)	0.93
ICU length of stay (days)	1 (0–16.5)	25 (18.5–34.5)	<0.01
CCI	0 (0–1)	1 (0–2)	0.14
Time to infection (days of PM)	10 (6.3–21.5)	17 (9–24)	0.49
Previous hospitalization	6	8	0.48
Previous antibiotic treatment	6	6	0.16
Previous head surgery	2	6	1

Abbreviations: CCI: Charlson Comorbidity Index, ESR: erythrocyte sedimentation rate, ICU: intensive care unit, PCT: procalcitonin, PM: postoperative meningitis.

## Data Availability

Anonymised study data are available upon request.
